# Mechanical behavior of coal and rock with central hole defects under eccentric loading conditions

**DOI:** 10.1038/s41598-025-02810-5

**Published:** 2025-05-26

**Authors:** Lina Ge, Jiajia Feng, Zhixuan Jia, Tao Wang

**Affiliations:** 1 School of Vehicle and Transportation Engineering, Taiyuan University of Science and Technology, Taiyuan, 030024 Shanxi People’s Republic of China; 2 School of Safety and Emergency Management Engineering, Taiyuan University of Technology, Taiyuan, 030600 Shanxi People’s Republic of China; 3 State Key Laboratory of Coal Mine Disaster Prevention and Control, Coal Technology and Engineering Group Shenyang Research Institute, Shenfu Demonstration Zone, Shenyang, 113122 People’s Republic of China

**Keywords:** Coal and rock, Eccentric loading, Mechanical behavior, Particle flow, Civil engineering, Materials for energy and catalysis, Coal

## Abstract

Coal and rock masses in mining projects are often subjected to varying degrees of eccentric loading. To analyze the effect of eccentric loading on the mechanical properties and damage evolution law of coal and rock containing hole defects, a biaxial compression test of coal and rock with eccentric loading was carried out with the help of PFC particle flow program. The results show that the peak stress of the coal and rock decreases linearly with the increase of the eccentricity coefficient under different circumferential pressures. The dominant crack pattern is influenced by the relative position of the hole defects to the loaded area. When the hole defects are located within the loaded area, the main control cracks formed pass through the hole defects, and when the hole defects are located outside the loaded area, the main control cracks do not pass through the hole defects. The evolution process of the number of microcracks in concrete under uniformly distributed load and eccentric load conditions can be divided into three stages: the calm period in the initial loading stage, the pre peak propagation period from the crack initiation point to the peak point, and the rapid increase period after the peak.

## Introduction

During the mining of coal resources, the coal and rock is often in a certain eccentric loading environment. For example, the coal and rock mass at the foot of the lower slope of the open-pit mine will be subjected to the eccentric load formed by the self weight of the overlying rock layer. The coal and rock mass in front of the mining face and the coal pillars on one side of the mining roadway will be affected by the supporting pressure and placed in an eccentric load environment^1–4^. The mining roadway of steeply inclined coal seams is affected by the self weight of the overlying rock layers and is also in an eccentric load environment^5,6^. Eccentric loads can exacerbate the damage and degradation process of coal and rock masses, leading to a tendency towards instability in spatial structures that can maintain stability under uniformly distributed loads. In addition, macroscopic or mesoscopic defects such as fractures and pores are commonly present in coal bodies due to the influence of diagenesis, cementation or external disturbances during mining processes, as well as stress redistribution. The existence of defects can lead to significant deterioration of mechanical parameters such as strength and elastic modulus of coal and rock masses^7,8^. Therefore, it is of great theoretical value to study the effects of the coupling of eccentric loading and primary defects on the mechanical behavior and crack initiation, expansion and penetration modes of coal and rock masses, and it is of great practical guidance for the determination of the dimensional parameters of coal pillars and the safe mining of coal resources.

Scholars have conducted extensive research on the mechanical behavior of rocks containing defects. Lee et al. studied the crack initiation, propagation, and coalescence modes at pre-existing open cracks or defects in three materials under uniaxial compression using the discrete element method^9^. Huang et al. studied the micromechanical response mechanism of crack propagation in intermittent double fractured red sandstone under different confining pressures through particle flow simulation^10^. Yang et al. used particle flow simulation to study the influence of fracture inclination angle on the mechanical parameters and crack propagation characteristics of double hole fracture specimens^11^. Tian et al. used the particle flow program PFC2D to study the fracture process of brittle sandstone with coplanar double fractures under different confining pressures^12^. J. Duriez et al. used the discrete element method to study the crack propagation of pre-existing defects with different properties in intact rocks^13^. However, in practical engineering, rock masses are often subjected to eccentric loads, and except for the surface of the mining space, the coal and rock underground are in a triaxial stress state. Conducting triaxial compression tests on coal and rock is also an essential means of studying the mechanical properties of underground coal and rock. Liu et al. conducted triaxial compression tests to study the deformation and failure characteristics of coal and rock^14–17^. Peng et al. and Meng et al. investigated the damage characteristics of coal and rock under triaxial compression using CT scanning and acoustic emission methods, respectively^18,19^. There are numerous research results on the mechanical behavior of coal and rock under triaxial compression, but few have considered the coupled effects of eccentric load triaxial compression and initial defects on the mechanical properties of rocks. Wang et al. studied the evolution characteristics of coal and rock damage under eccentric load^20^. Zang and Yoon studied the influence of eccentric triaxial compression on the brittle failure characteristics of granite^21,22^. However, the above studies were conducted on complete specimens and did not take into account the coupling effect of eccentric loads and defects. Wang et al. investigated the deformation and damage characteristics of coal and rock with pore defects under uniaxial compression asymmetric loads, but did not consider the influence of confining pressure^23^.

In view of this, this article conducted a particle flow simulation study on the mechanical behavior of coal and rock with pore defects under eccentric load biaxial compression. The research results are of great significance for revealing the mechanism and control technology of asymmetric deformation and instability caused by eccentric loads in engineering practice. The research results are of great significance for revealing the asymmetric deformation and instability mechanism of roadway caused by eccentric load and the proposal of control technology.

## Particle flow simulation scheme

The PFC2D particle flow program is a two-dimensional discrete element software commonly used to simulate rock mechanics behavior. The particles in the rock sample are simulated using rigid disks, and the connections between particles are simulated using parallel bonding. This program can track the initiation and propagation process of cracks in real time, and distinguish between tensile and shear cracks, which is widely used in the study of jointed rock masses. Previous research has shown that in the PFC discrete element calculation method, two-dimensional numerical simulation results can well reflect the mechanical properties and damage and failure laws of coal and rock. Therefore, this article adopts a two-dimensional plane stress model to study the mechanical properties and damage evolution laws of coal and rock samples with pore defects under eccentric load^24,25^.

### Establishment of numerical calculation model

**Fig. 1:**
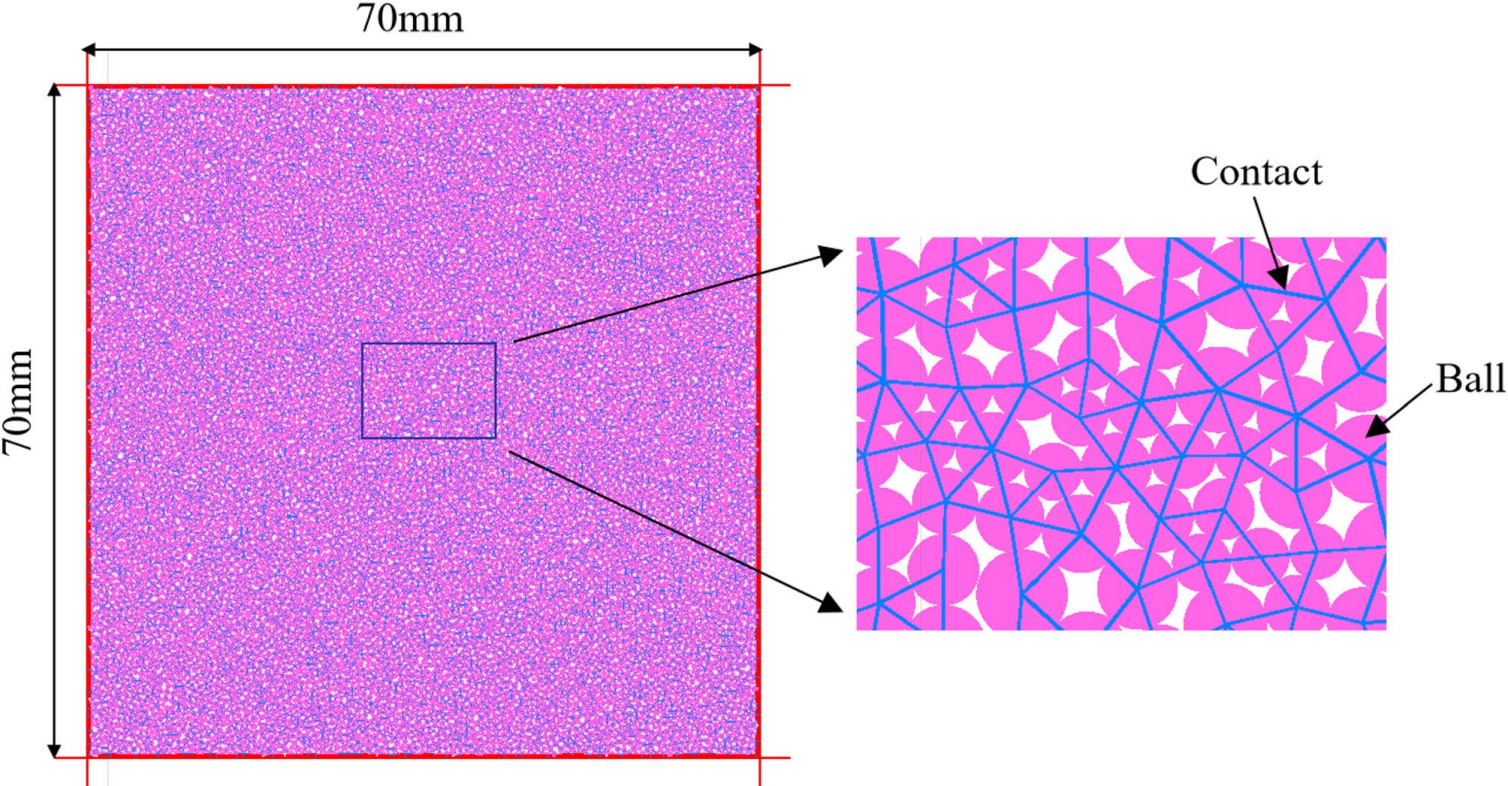
Model of numerical calculation.

The model established by numerical simulation is consistent with the sample size used in indoor experiments, both of which are cubic samples with a side length of 70 mm. The minimum particle diameter is 0.2 mm, the particle size ratio is set to 1.66, the particles are randomly and uniformly distributed, and the number of particles is 16,724. The parallel bonding model is adopted between particles, and the model is established as shown in Fig. 1.

### Calibration of mesoscopic parameters

**Fig. 2:**
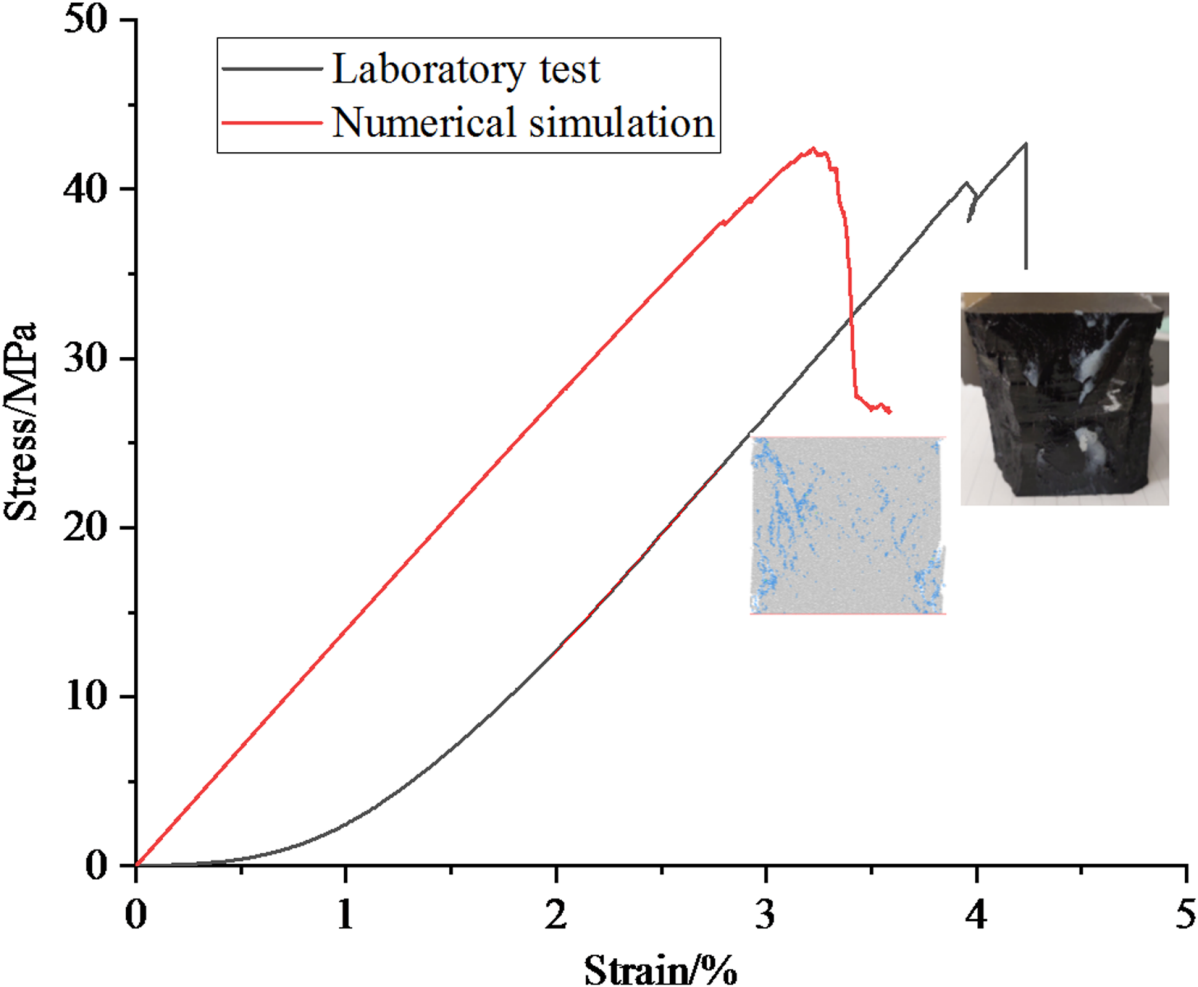
Stress strain curves of indoor experiments and numerical simulations.

Raw coal lumps taken from the project site were transported to the laboratory and made into cubic specimens with a side length of 70 mm through cutting and grinding processes. Then uniaxial compression test was carried out on the complete raw coal samples to obtain the macroscopic mechanical parameters of the coal samples. The “trial and error method” is used to calibrate the mesoscopic parameters based on the uniaxial compression test results of complete raw coal samples^26–28^. If the mechanical parameters and macroscopic failure modes obtained from numerical simulation are not significantly different from the indoor test results by continuously adjusting the values of the mesoscopic parameters, it can be considered that the calibration of the mesoscopic parameters is successful. The stress-strain curves and failure modes of the specimens obtained from numerical simulation and indoor experiments are shown in Fig. 2, and the macroscopic mechanical parameters of coal rock are shown in Table 1. According to Fig. 2; Table 1, the peak stress, elastic modulus, and Poisson’s ratio of coal rock obtained from numerical simulation and indoor testing are not significantly different, which indicates that the calibration of mesoscopic parameters in particle flow simulation has been successful, and the successfully calibrated mesoscopic parameters are shown in Table 2.

**Table 1:** Mechanical parameters of coal and rock obtained from laboratory test and numerical simulation.

Item	Peak stress(MPa)	Elastic modulus(GPa)	Poisson’s ratio
Test result	42.69	1.42	0.21
Simulation results	42.40	1.38	0.23
Deviation	0.68%	2.8%	8.6%

**Table 2:** Micromechanical parameters used in PFC2D simulation.

Mesoscopic parameters	Value
The minimum particle radius *R*_min_/m	2.1 × 10^− 4^
Ratio of maximum to minimum particle radius	1.66
Particle density *ρ*/(kg/m^3^)	2700
Friction coefficient between particles *µ*	0.3
Elastic modulus between particles *E*_*c*_/GPa	1.4
Contact bonding stiffness ratio kn/ks	1.8
Parallel bonding radius coefficient *λ*	1
Elastic modulus of parallel bonding *E*_*c*_/GPa	1.4
Parallel bonding stiffness ratio ${\overline {k} _n}/{\overline {k} _s}$	1.8
porosity	0.14
Parallel bonding tensile strength ${\overline {\sigma } _c}$/MPa	27
Parallel bonding cohesion $\overline {c}$/MPa	33
Parallel bonding internal friction angle $\overline {\varphi }$/(°)	30

## Simulation of particle flow in biaxial compression of coal and rock with hole defects under eccentric load

### Simulation scheme for eccentric load biaxial compression

**Fig. 3:**
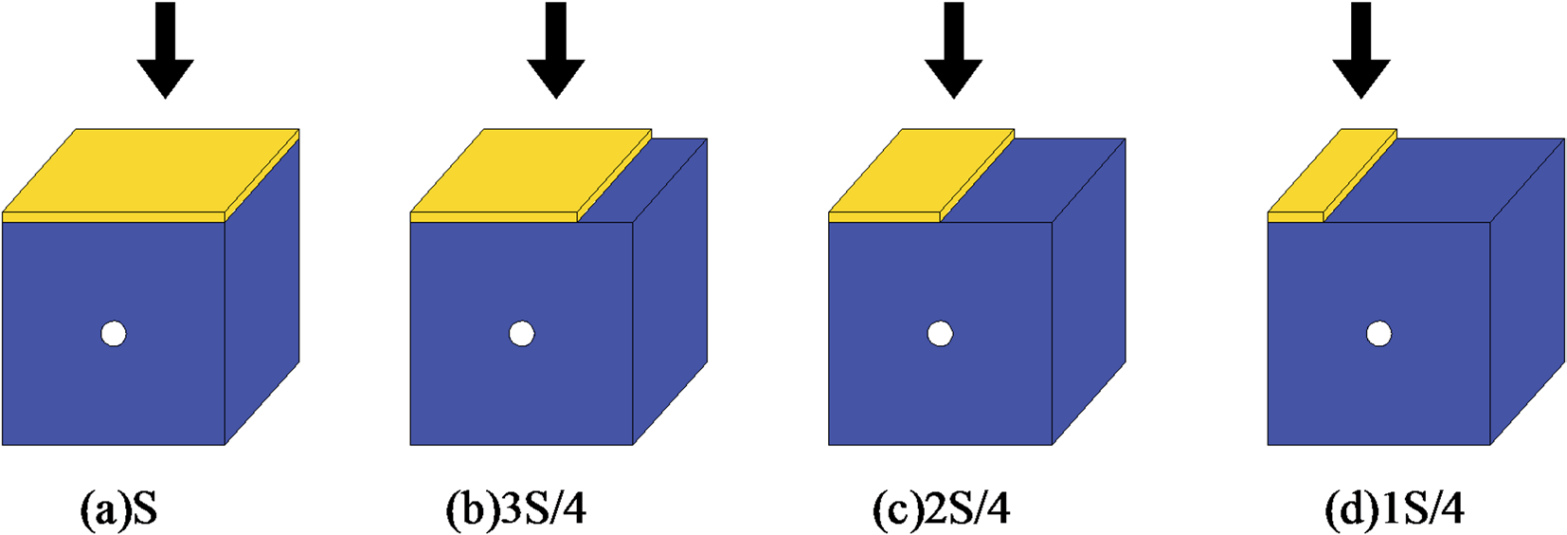
Schematic diagram of eccentric load loading.

As shown in Fig. 3, the eccentric load is an axial local load or asymmetric load, the load eccentricity coefficient is defined to represent the degree of load eccentricity, which is the ratio of the non loading area to the loading area.

1 $${I_{\text{c}}}{\text{=1-}}\frac{{{{\text{S}}_{\text{f}}}}}{{\text{S}}}$$

In the formula, *I*_c_ is the load eccentricity coefficient, S_f_ is the loading area, and S is the surface area of the sample.

According to Eq. (1), when the loading areas are 1 S/4, 2 S/4, 3 S/4, and S respectively, the corresponding load eccentricity coefficients are 0.75, 0.5, 0.25, and 0.

**Fig. 4:**
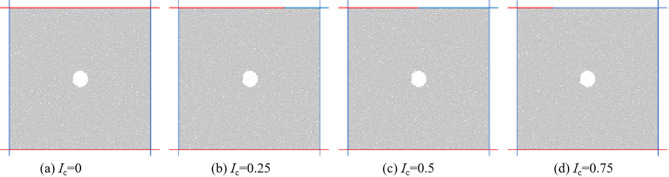
Simulation scheme of biaxial compression under eccentric load.

When the eccentric load is subjected to biaxial compression, the loading of confining pressure and axial pressure is achieved through the movement of the wall. The function of the wall under different degrees of eccentric load loading is shown in Fig. 4. The red wall in Fig. 4 is the loading wall, which applies axial load to the specimen through the relative movement of the upper and lower walls. The blue wall is a servo wall, which applies varying degrees of confining pressure to the sample. This article conducted biaxial compression tests under uniformly distributed loads and three different degrees of eccentric loads. The moving speed of the loaded wall during the experiment is set to 0.05 m/s, which meets the requirements of static loading.

### Mechanical properties of coal and rock under eccentric load biaxial compression

**Fig. 5:**
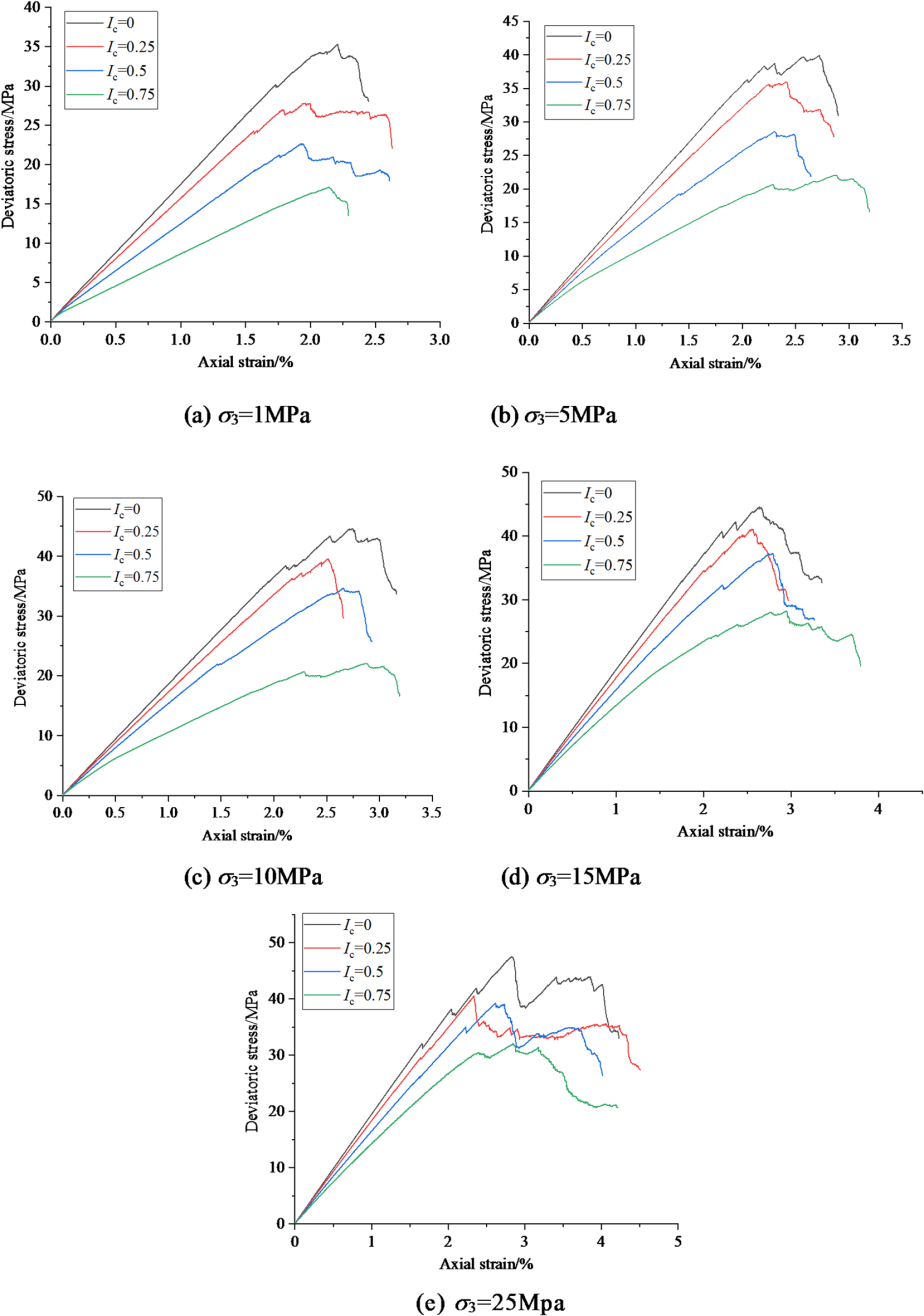
Biaxial compressive stress-strain curves under eccentric load under different confining pressures.

The stress-strain curves of coal and rock under eccentric load compression under different confining pressures are shown in Fig. 5.

**Fig. 6:**
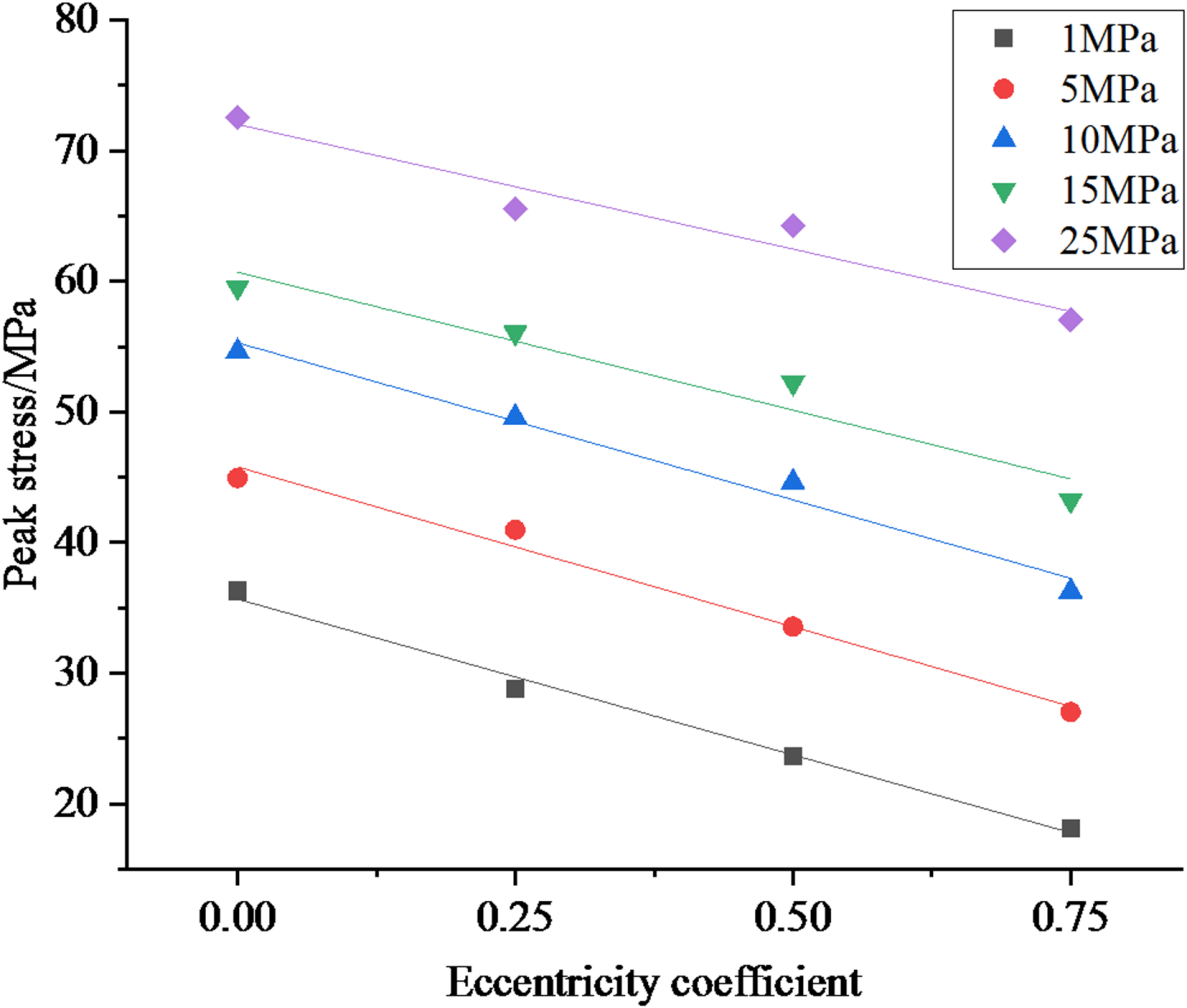
The relationship curve between peak stress and eccentricity coefficient.

As shown in Fig. 5, under different confining pressures, the peak stress of coal and rock gradually decreases with the increase of axial load eccentricity coefficient. As the confining pressure increases, the post peak plastic flow phenomenon of coal and rock becomes more pronounced, and the strain value required to decrease to the same stress level becomes higher. Figure 6 shows the relationship between peak stress and eccentricity coefficient under different confining pressures. According to Fig. 6, under different confining pressures, as the eccentricity coefficient increases, the peak stress of coal and rock gradually decreases, and the two are approximately linearly related.

### Analysis of failure mode of coal and rock under eccentric load biaxial compression

**Fig. 7:**
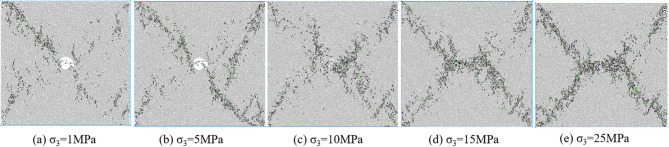
Biaxial compression failure mode when *I*_c_=0.

**Fig. 8:**
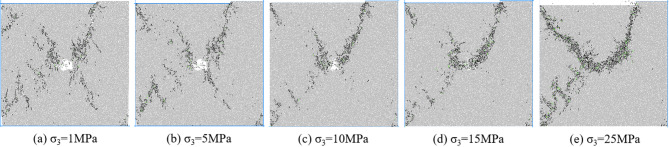
Biaxial compression failure mode when *I*_c_=0.25.

**Fig. 9:**
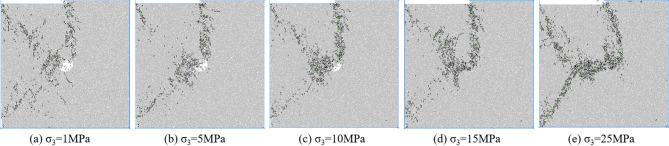
Biaxial compression failure mode when *I*_c_=0.5.

**Fig. 10:**
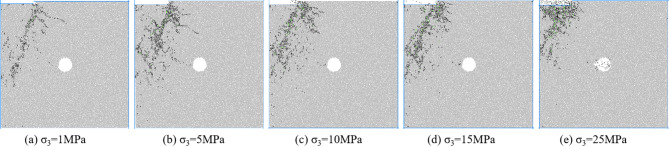
Biaxial compression failure mode when *I*_c_=0.75.

Figures 7, 8, 9 and 10 shows the failure modes of coal and rock at 80% σ_c_ after the peak value under different degrees of eccentric load. As shown in Fig. 7, under uniformly distributed loads, coal and rock undergo X-shaped shear failure under different confining pressures, and the conjugate shear cracks formed are all penetrated by pore defects. Mesoscopic failure cracks are mainly concentrated in the X-shaped shear failure zone, and with the increase of confining pressure, most of the fractures after failure are tensile cracks, and the macroscopic shear failure caused by mesoscopic tensile cracking occurs in coal and rock.

Figure 8 shows the macroscopic failure mode of coal and rock with an eccentricity coefficient of 0.25 under different confining pressures. According to Fig. 6, under different confining pressures, the mesoscopic cracks during coal and rock failure are distributed near the load zone and shear zone, and there are basically no mesoscopic cracks in the non load zone. At low confining pressure, macroscopic cracks connect to pore defects and basically penetrate the shear band between the load zone and the non load zone. As the confining pressure increases, mesoscopic cracks no longer appear on the lower right side of the hole defect. Mesoscopic cracks are concentrated in the area connecting hole defects and the end of the loading plate. From low confining pressure to high confining pressure, coal and rock transition from near X-type failure to near Y-type failure.

Figure 9 shows the macroscopic failure mode of coal and rock with an eccentricity coefficient of 0.5 under different confining pressures. According to Fig. 9, the failure mode of coal and rock with an eccentricity coefficient of 0.5 is basically the same as that with an eccentricity coefficient of 0.75. Mesoscopic cracks are distributed in the load zone and the concentrated shear stress zone, and there is basically no distribution of mesoscopic cracks in the non load zone. The hole defect is located directly below the shear stress concentration zone. The main control crack formed by coal and rock failure is located in the shear stress concentration zone, connecting the end of the loading plate with the hole defect. The final crack morphology formed is a semi X-shaped crack in the load zone and an I-shaped crack in the stress concentration zone, which converge at the location of the hole defect. The types of mesoscopic cracks are mostly tensile cracks, and the macroscopic shear failure caused by mesoscopic tensile cracking occurs in coal and rock.

Figure 10 shows the macroscopic failure mode of coal rock with an eccentricity coefficient of 0.75 under different confining pressures.

According to Fig. 10, when the eccentricity coefficient is 0.75, the hole defect is located outside the load zone, and the main control crack formed by coal and rock failure does not pass through the hole defect. The main control crack is located between the load zone and the non load zone, and deviates towards the side of the specimen. As the confining pressure increases, the length of the main control crack gradually decreases, and the distribution of mesoscopic cracks concentrates towards the loading plate area. Most mesoscopic cracks are tensile cracks, while coal and rock undergo macroscopic shear failure caused by mesoscopic tensile cracking.

From the above analysis, it can be concluded that during biaxial compression under eccentric load, the dominant crack morphology during coal and rock failure is influenced by the relative position between the pore defects and the load zone. When the hole defect is located within the load zone, the main control crack formed passes through the hole defect. When the hole defect is located outside the load zone, the increase in load eccentricity weakens the influence of the hole defect on the morphology of the main control crack, and the main control crack does not pass through the hole defect.

3.4 Study on the influence of eccentric load on micro cracks in coal and rock under biaxial compression.

**Fig. 11:**
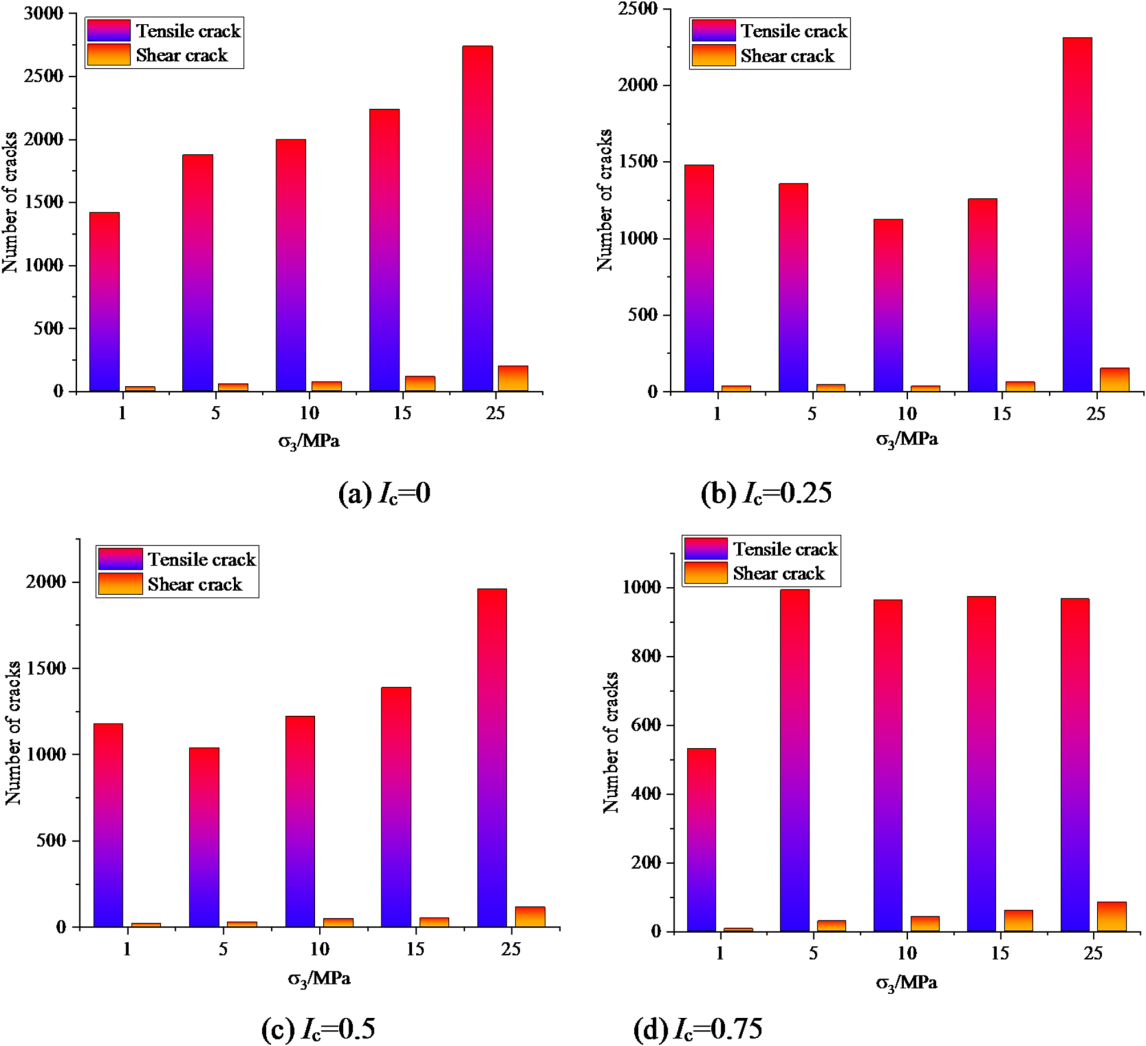
The number of cracks in coal and rock after failure under different eccentricity coefficients.

Figure 11 shows the number of cracks when the post peak stress of coal and rock drops to 80%σ_c_ under different eccentricity coefficients.

As shown in Fig. 11, when *I*_c_=0, i.e. under uniformly distributed load, the number of tensile and shear cracks in coal and rock gradually increases with the increase of confining pressure, and the number of tensile cracks is much greater than the number of shear cracks, indicating that the greater the confining pressure, the more severe the coal and rock damage. When *I*_c_=0.25, *I*_c_=0.50 and *I*_c_=0.75, the number of tensile cracks after coal and rock failure did not show a consistent pattern with the increase of confining pressure. However, the number of tensile cracks did not differ significantly at different confining pressures, and this feature was more pronounced at *I*_c_=0.75. When subjected to eccentric load, the number of mesoscopic shear cracks gradually increases with the increase of confining pressure.

**Fig. 12:**
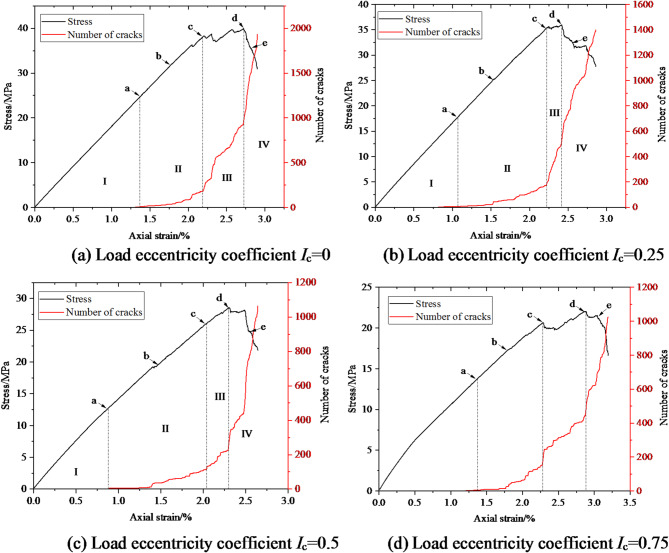
Evolution curve of crack number and strain under different load eccentricity coefficients.

Taking the confining pressure of 5 MPa as an example, Fig. 12 shows the evolution curve of the number of coal and rock cracks under biaxial compression with different degrees of eccentric load. As shown in Fig. 12, the number of cracks increases continuously with the increase of axial strain during biaxial compression under eccentric load. According to the evolution curve of crack number, it can be divided into four stages: the calm stage I before crack initiation, the stable crack propagation stage II, the unstable crack propagation and penetration stage III, and the post failure stage IV.

**Fig. 13:**
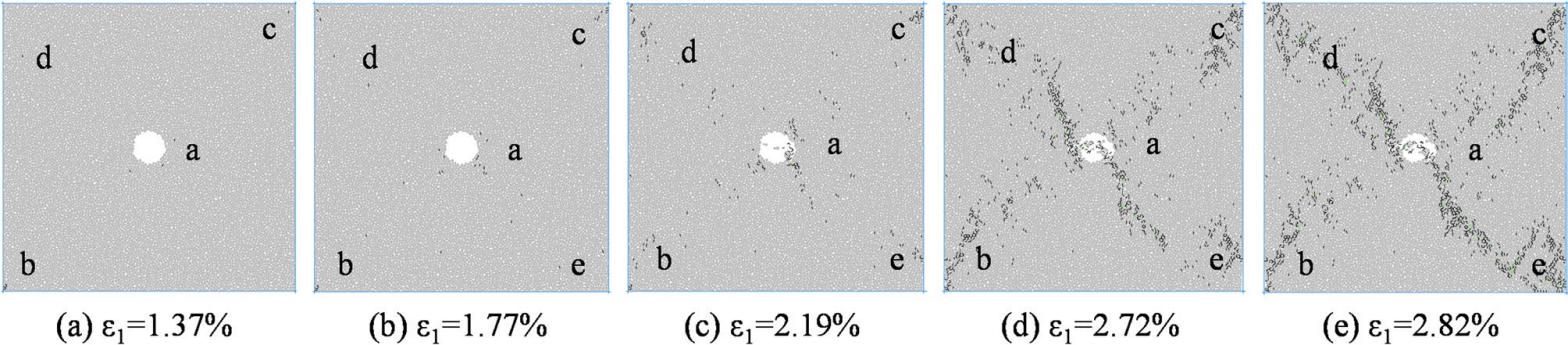
Meso crack propagation and evolution process when *I*_c_=0.

**Fig. 14:**
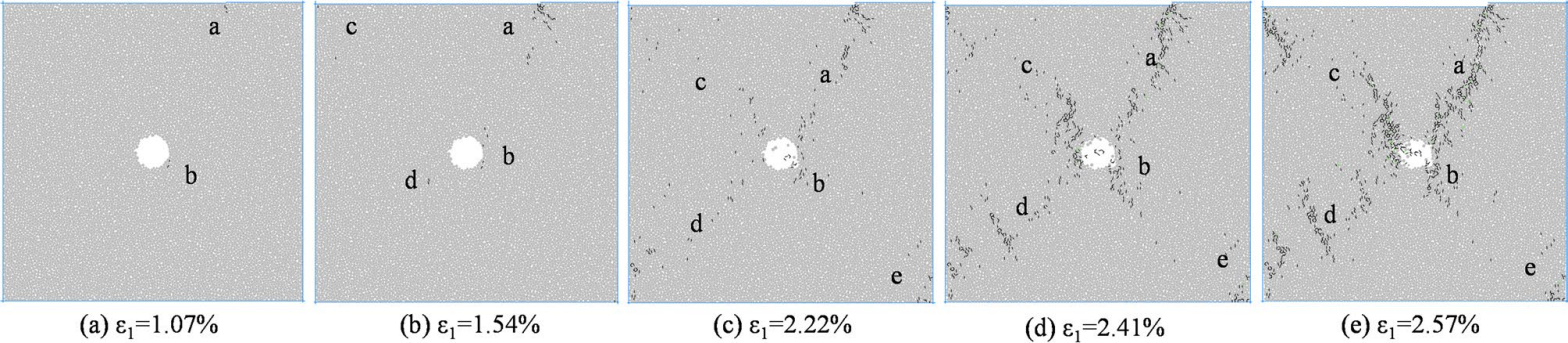
Meso crack propagation and evolution process when *I*_c_=0.25.

**Fig. 15:**
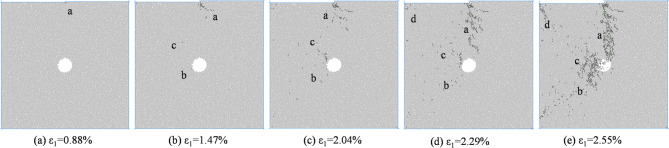
Meso crack propagation and evolution process when *I*_c_=0.5.

**Fig. 16:**
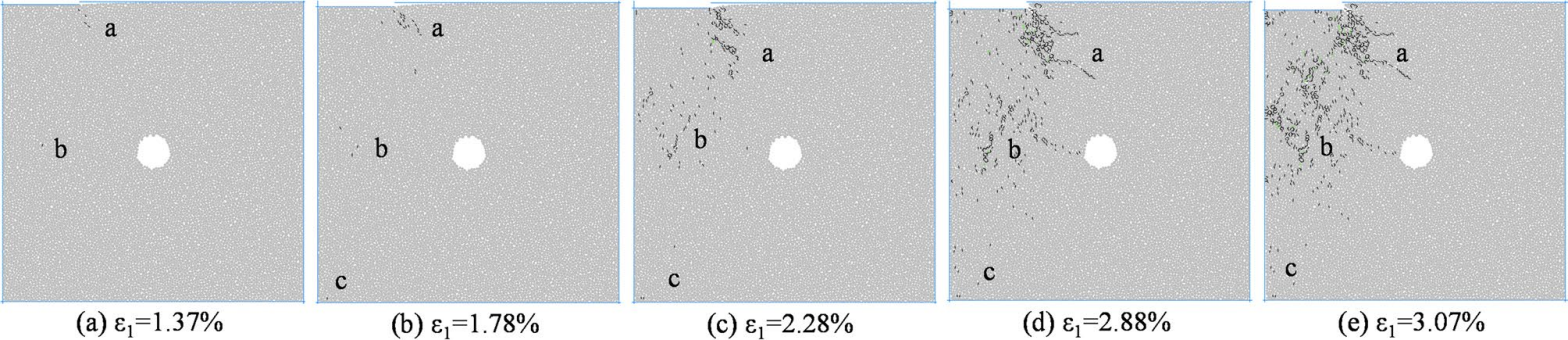
Meso crack propagation and evolution process when *I*_c_=0.75.

In order to analyze the initiation and propagation evolution process of micro cracks in coal and rock at different loading stages, typical loading moments were selected in Fig. 12, and the distribution positions of micro cracks at different typical loading moments were given, as shown in Figs. 13, 14, 15 and 16.

From Fig. 13, it can be seen that under uniformly distributed load, cracks first initiate around the defect a and at the corners b, c, and d of the specimen, and as the axial strain increases, new cracks continue to appear near the above-mentioned area, intermittently appearing in the diagonal area of the specimen, with the most concentrated defects around the holes. At the peak moment, mesoscopic cracks are interconnected and concentrated in the diagonal area of coal and rock, forming X-shaped macroscopic failure cracks. The number of cracks after the peak continues to increase, but the distribution location has not changed, mainly concentrated in the diagonal area of coal and rock.

From Fig. 14, it can be seen that when *I*_c_=0.25, the mesoscopic crack first starts from the stress concentration position around the loading plate end and the hole defect. The crack direction in area a is approximately parallel to the loading direction, while the crack direction in area b is approximately parallel to the tangent direction of the hole. As the axial strain increases, the number of cracks in region a gradually increases and extends towards the direction of the hole defect. Cracks in region b around the hole defect increase and extend towards region a, while cracks appear in region d near the hole and region c at the end of the specimen. As the strain further increases, the number of cracks in regions a, b, c, and d increases, showing a trend of penetration, and new cracks appear in region e. At the peak stress moment, cracks in regions a, b, and d interconnect, causing damage to the specimen. The number of cracks in the a, b, c, and d regions after the peak further increased, while the number of cracks in the e region did not show a significant change.

From Fig. 15, it can be seen that when *I*_c_=0.5, the mesoscopic crack first starts from the stress concentration area at the end of the loading plate. As the axial strain increases, cracks propagate towards the direction of the hole defect, and new cracks appear around the hole defect. As the axial strain further increases, the number of cracks around the hole defect increases, and new cracks appear at both ends of the specimen, with the number of cracks gradually increasing. At the peak stress moment, cracks in regions a and c interconnect, causing the specimen to rupture. As the axial strain continues to increase, the number of cracks in regions a and c gradually increases, and the cracks in regions b and c show a trend of penetration. From the distribution of cracks after coal and rock failure, it can be seen that the left side of the hole defect is located in the load zone, and the number of cracks is significantly higher than that on the right side of the hole defect.

According to Fig. 16, when *I*_c_=0.75, mesoscopic cracks first appear in the stress concentration area at the end of the loading plate. With the increase of axial strain, new cracks are generated in the region b near the stress concentration zone, and then the cracks gradually accumulate and propagate in regions a and b, and new cracks are generated in the region c of the load action zone at the end of the specimen. At the moment of peak stress, the cracks in regions a and b are interconnected, causing damage to the specimen. In the entire load zone, the distribution of upper cracks is basically uniform, and region a is the most concentrated. After peak stress, cracks further appear in regions a and b, and the damage to the specimen becomes more severe.

## Conclusion

1. Under different confining pressures, as the eccentricity coefficient of axial load increases, the peak stress of coal and rock gradually decreases. As the confining pressure increases, the post peak plastic flow phenomenon of coal and rock becomes more pronounced, and the strain value decreases to the same stress level and becomes higher. Under different confining pressures, as the eccentricity coefficient increases, the peak stress of coal and rock gradually decreases, and the two are approximately linearly related.
2. When subjected to eccentric biaxial compression, the shape of the main controlled crack during coal and rock failure is influenced by the relative position of the hole defect and the load zone. When the hole defect is located within the load zone, the main control crack formed passes through the hole defect. When the hole defect is located outside the load zone, the increase in load eccentricity weakens the influence of the hole defect on the morphology of the main control crack, and the main control crack does not pass through the hole defect.
3. When subjected to eccentric biaxial compression, the evolution of the number of mesoscopic cracks can be divided into four stages: the calm stage I before crack initiation, the stable crack propagation stage II, the unstable crack propagation and penetration stage III, and the post failure stage IV.
4. During the fracture process of coal and rock under biaxial compression with eccentric load, the number of mesoscopic tensile cracks is much greater than that of mesoscopic shear cracks. Under different degrees of eccentric load, the failure mode of coal and rock is macroscopic shear failure caused by mesoscopic tensile cracks.

## Data Availability

The datasets used or analyzed during the current study are available from the corresponding author on reasonable request.
